# Read the clonotype: Next-generation sequencing-based lymphocyte clonality analysis and perspectives for application in pathology

**DOI:** 10.3389/fonc.2023.1107171

**Published:** 2023-02-08

**Authors:** Patricia J. T. A. Groenen, Michiel van den Brand, Leonie I. Kroeze, Avital L. Amir, Konnie M. Hebeda

**Affiliations:** ^1^ Department of Pathology, Radboud University Medical Center, Nijmegen, Netherlands; ^2^ Radboud Institute for Molecular Life Sciences, Radboud University Medical Center, Nijmegen, Netherlands; ^3^ Pathology-DNA, Location Rijnstate Hospital, Arnhem, Netherlands

**Keywords:** immunoglobulin E, T-cell receptor, gene rearrangement, clonality assessment, NGS, pathology

## Abstract

Clonality assessment using the unique rearrangements of immunoglobulin (IG) and T-cell receptor (TR) genes in lymphocytes is a widely applied supplementary test for the diagnosis of B-cell and T-cell lymphoma. To enable a more sensitive detection and a more precise comparison of clones compared with conventional clonality analysis based on fragment analysis, the EuroClonality NGS Working Group developed and validated a next-generation sequencing (NGS)-based clonality assay for detection of the IG heavy and kappa light chain and TR gene rearrangements for formalin-fixed and paraffin-embedded tissues. We outline the features and advantages of NGS-based clonality detection and discuss potential applications for NGS-based clonality testing in pathology, including site specific lymphoproliferations, immunodeficiency and autoimmune disease and primary and relapsed lymphomas. Also, we briefly discuss the role of T-cell repertoire of reactive lymphocytic infiltrations in solid tumors and B-lymphoma.

## 1 Introduction

Assessment of the clonality of the immunoglobulin (IG) and/or T-cell receptor (TR) genes is an important aid in the diagnosis of lymphoproliferative diseases. Lymphoma cells originate from a single transformed lymphoid cell and therefore all malignant cells have the same IG gene rearrangements in B-cell lymphoma (BCL) or TR rearrangements in T-cell lymphoma (TCL). Clonality testing uses this feature and facilitates the discrimination between clonally expanded cells and reactive cells with diverse IG and/or TR rearrangements. The BIOMED-2/EuroClonality assays for clonality testing have been used worldwide for more than a decade now. The strength of these assays is the complementarity of different PCR targets, which results in an unprecedented high detection rate (1–4).

Clinical clonality assessment is mainly used when discrimination between a lymphoma and a reactive lymphoid infiltrate is uncertain. Examples are low-grade lymphoma, including Bcl2-negative follicular lymphomas (5), B- or T-cell proliferations at specific sites, such as the skin, or in the context of immunodeficiency. Also lymphoproliferations with a combination of atypical B- and T-cells, such as angioimmunoblastic TCL, or lymphomas of unclear lineage, like some anaplastic large cell lymphoma, can benefit from clonality studies. In case of small biopsies clonality assessment can guide further diagnostics. It is, however, not always suitable to detect Hodgkin lymphoma, as the traditional EuroClonality/BIOMED-2 protocols may not be sufficiently sensitive to detect small numbers of malignant cells in a background of lymphoid cells, as is the case in most classical Hodgkin lymphomas (6–8). Also, it is important to note that lineage determination in acute lymphoblastic leukemia cannot be based on clonality assays alone, since neoplastic precursor B and T cells often show cross-lineage rearrangements (9). In addition, monoclonality does not always imply malignancy since some reactive processes contain clonal lymphocytic populations (10).

## 2 NGS-amplicon based clonality assessment

**Figure 1:**
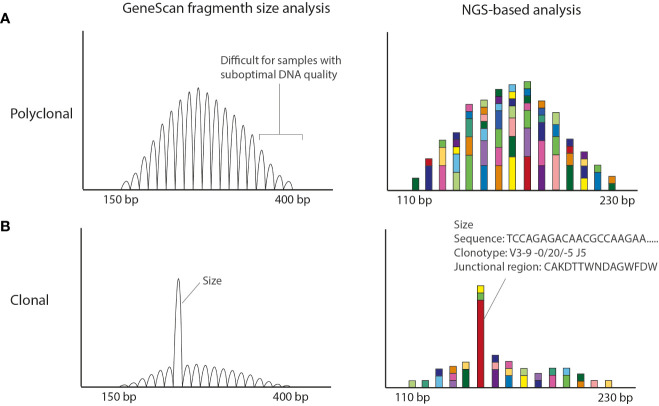
Conventional EuroClonality/BIOMED-2 fragment size vs NGS-amplicon based clonality analysis. **(A)** Polyclonal pattern observed in Genescan and NGS-based analysis. The size of the amplicons is smaller in the NGS-based approach making the technique more suitable for smaller DNA fragments obtained from FFPE material. **(B)** Clonal result (with low polyclonal background). Using GeneScan analysis only the size of the rearrangement is known, whereas with NGS-amplicon based clonality also the nucleotide sequence is obtained. The Bioinformatic program ARResT/Interrogate processes the nucleotide sequences into clonotypes, in which the used V, D and J genes as well as the junctional region in amino acids are defined.

For the conventional clonality assays multiple IG targets, notably IGHV-IGHD-IGHJ (in FR 1,2,3), IGHD-IGHJ, IGKV-IGKJ, IGKV-KDE and Intron RSS-KDE, are analyzed. Likewise, TRGV-TRGJ, as well as TRBD-TRBJ and TRBV-TRBD-TRBJ are assessed for T-cell clonality (1). A polyclonal population harboring different V(D)J rearrangements gives rise to a range of differently sized PCR, resulting in a Gaussian distribution in fragment analysis (GeneScan) (**Figure 1A**). In case of a clonal population, there will be one or two dominant PCR products of a given size per target with GeneScan (**Figure 1B**). Guidelines for the uniform technical scoring of the individual PCR targets and the molecular conclusion of the entire sample, which is deduced from the results of the individual targets, have been developed (11). Finally, the result of the clonality assessment has to be interpreted in the context of the clinical presentation, other molecular studies, and the pathological findings.

Despite the good performance and the world-wide usage of these assays, they have some weaknesses that potentially yield (mainly) false-negative results. The BIOMED-2/EuroClonality assays have been designed for high-quality DNA samples generating amplicons in the range of 150–400 bp (1) (**Figure 1**). However, formalin-fixed paraffin-embedded (FFPE) tissue specimens generally used in a diagnostic setting, are affected by DNA crosslinking and fragmentation, resulting in DNA samples of suboptimal quality with short DNA fragments. Clonal rearrangements with longer amplicons may potentially go unnoticed in samples of suboptimal DNA quality. In addition, the detection of a small clonal rearrangement in a background of polyclonal B-cells is dependent on the position of the clonal product within the Gaussian curve; it can be entirely hidden in the polyclonal background. Furthermore, for clonal comparison only the size of the PCR fragments is used (**Figure 1**). Since different rearrangements may result in the same size PCR fragments, this may hamper interpretation, especially in cases in which a single rearranged target is detected.

To tackle these issues, the EuroClonality NGS-working group has developed next-generation sequencing (NGS)-based clonality assays for detection of IG and TR gene rearrangements (12, 13), which can be analyzed with the bioinformatics tool ARResT/Interrogate (14). They are based on the use of gene-specific primers and importantly, on the generation of shorter amplicon sizes, which makes them more suitable for clonality detection in samples of suboptimal DNA-quality, like FFPE-samples. NGS-based clonality assays provide the nucleotide sequences of all IG and/or TR rearrangements, both from the malignant lymphoid cells and from the non-malignant background cells (**Figure 1**).

**Figure 2:**
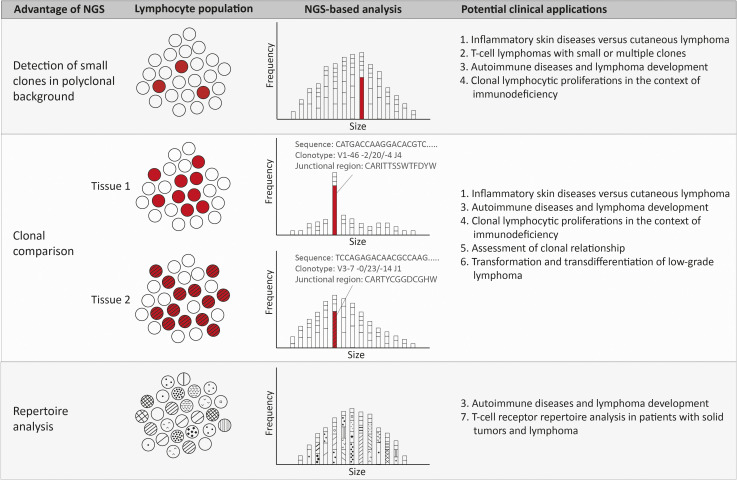
Advantages and potential clinical applications of NGS-amplicon based clonality analysis. Using NGS-amplicon based clonality analysis, small clones in a polyclonal background, or even hidden in the polyclonal background, can be more confidently detected. A second advantage of NGS is the sequence information and therefore the clonotype information, which is very useful for rapid and reliable clonal comparison. The clonotype obtained from the nucleotide sequence reveals the V, (D) and J gene as well as the junctional region that allows quick and efficient comparison of clonotypes of two or more specimen. Suspected ongoing somatic hypermutation in one of the specimens requires in depth investigation of the nucleotide sequences. The clonotypes also enable detection of the repertoire of the lymphocytes, including early detection of possibly malignant clonotypes.

Bioinformatic software analyzes each sequence for the presence of V, (D) and J genes and the junctional region containing the complementary determining region 3, which is represented as an amino acid sequence. With this information the sequences are attributed to clonotypes. A clonotype is characterized by the same V and J gene and junctional region. Therefore reliable detection of minor clonal rearrangements is possible, resulting in a high sensitivity (12, **Figure 2**).

NGS-based IG clonality assessment showed a very good performance in a cohort of BCL, the vast majority of which were FFPE samples (15). In diagnostic FFPE samples of classical Hodgkin lymphoma (cHL), NGS-based detection of IG rearrangements was more accurate and sensitive to detect clonal rearrangements compared to the conventional BIOMED-2/EuroClonality assay (16). Because of the sequence information and the high sensitivity, the NGS-based clonality assays are very valuable to solve complex rearrangement patterns (17). They allow detailed comparison of sequential lesions or multiple lymphomas at different locations within a patient (18). The technical improvements due to the advent of NGS will allow higher sensitivity, more detailed analysis and broader applications, as will be discussed below.

## 3 Potential clinical applications

In various clinical situations the advantages of NGS-based clonality analysis can be exploited (**Figure 2**).

### 3.1 Inflammatory skin diseases

One important use of clonality analysis for the differentiation between inflammatory disease and lymphoma is in the diagnostic workup of mycosis fungoides (MF), the most common cutaneous TCL (19, 20) with a rising incidence (21). The diagnosis of MF can be difficult due to morphological overlap with various inflammatory skin diseases (ISD). Especially the early-stage of MF is both clinically and histologically difficult to distinguish from ISDs such as eczema, psoriasis and cutaneous lupus erythematosus (19, 20, 22–24), resulting in a time to diagnosis of 3 to 4 years after the first lesions appear.

Immunohistochemistry can be very helpful in the diagnosis of MF, but is not always decisive. When the diagnosis of MF is uncertain based on clinical picture, histology and immunohistochemistry, TR clonality assessment can be used to help discriminate between MF and ISDs. However, especially in early-stage MF, the lesions often contain a relatively small number of neoplastic T-cells admixed with a relatively high number of polyclonal reactive T-cells. In the conventional clonality assay a small monoclonal population can be difficult or impossible to detect in a high polyclonal background, potentially hampering the diagnosis of early-stage MF (25–27). Since biopsies of ISDs can also contain a clonal population in a polyclonal background (28), the ultimate proof of MF is considered to be the presence of an identical dominant clonal population in two biopsies derived from different anatomical locations (29). In the conventional clonality assay, comparison of the clonality between two different samples is based on the fragment size of the detected clonal peak, which can be challenging and does not always represent the same rearrangement.

NGS-based clonality analysis can most likely overcome these obstacles, since the sequence information of the TR gene rearrangements can be used to identify small relevant clones amidst a polyclonal background and may identify early stage disease (30, 31). Another advantage of analyzing the sequences is comparison of the clones in biopsies from different lesions and multiple time points for identification of recurrent MF (30). The high sensitivity of NGS-based clonality also carries a risk of false-positive results. Therefore studies are warranted to determine the cut-off points that separate MF from reactive conditions before NGS-based clonality can be introduced in the diagnostic workup of suspected MF.

### 3.2 T-cell lymphomas

The diagnosis of TCL by histology is often straightforward, but certain TTCL subtypes regularly cause diagnostic problems. The above mentioned cutaneous lymphomas are one example, but another example is the group of TCL that are derived from CD4 positive follicular helper T-cells that normally are resident in germinal centers (32). This group includes angioimmunoblastic TCL (AITL), the most frequent nodal mature TCL in elderly patients in western countries (33). AITL is clinically characterized by immune dysregulation and autoimmunity related symptoms. Histology usually shows lymph nodes with a prominent reactive background of CD4 and CD8 positive T-cells and proliferating follicular dendritic cell meshworks, accompanied by a variable proliferation of B- and plasma cells, often EBV-driven (32, 34). In this situation a T-cell clone combined with a B-cell clone is frequently seen (35). In case of a small T-cell clone or several T- and B-cell clones, which is in our experience and according to literature (36) not uncommon in AITL, NGS-based tests will be more informative than conventional clonality analysis, since NGS can better identify small clones that are hidden in a polyclonal background of T-cells. Bi-clonal and oligoclonal AITL were identified in a significant number of cases in a study applying NGS-based clonality testing, reflecting AITL evolution from a mutated hematopoietic progenitor pool and the subsequent exposure to the complex and dynamic environment of the germinal center (37). This knowledge of the biology of AITL can help to unravel the composition of the infiltrate and support the diagnosis in challenging cases of AITL.

### 3.3 Autoimmune diseases

Autoimmune diseases are associated with a significantly increased risk of lymphoma development, as reviewed recently (38). One group with a very high risk is patients with Sjögrens syndrome (SjS), who suffer from chronic lymphoid infiltration in the salivary glands, causing destruction of glandular structures leading to atrophy. In this context, biopsies are notorious for the difficult discrimination between lymphoepithelial sialadenitis and the onset of marginal zone lymphoma (39). Indeed, the ectopic formation of lymphoid tissue with functional germinal centers in the salivary glands is related to both Sjs disease activity, and the risk for lymphoma development (40). But the mere detection of clonal B-cell proliferations in salivary gland biopsies does not correlate with the subsequent development of BCL, as was shown in a retrospective series of 49 Sjs patients with [n=21] or without [n=28] lymphoma development (41). 18% of the patients without subsequent BCL development showed clonal Ig rearrangements in the minor salivary glands by conventional clonality analysis.

More promising is the analysis of the IG repertoire in the inflamed tissues by NGS-based clonality analysis. This gives insight in the composition of the infiltrate and enables quantification of large numbers of IG clonotypes, including usage of V and J genes. Mainly high-affinity stereotypic rheumatoid factor producing B-cells from the autoreactive, often oligoclonal to polyclonal environment, show clonal evolution to BCL (42–44). Early detection and monitoring of these clones in the background of inflammation, by NGS-based clonality testing, is expected to be useful in the management of Sjs patients at high risk of lymphoma development.

### 3.4 Immunodeficiency

Lymphoid proliferations occurring in the background of immunodeficiency (ID) can involve virtually all tissues and range from benign lymphoid proliferations to full-blown lymphomas (45, 46). The main cause is T-cell suppression or dysfunction, caused by a germline defect in primary ID or induced by infection or therapy in secondary ID. Because of the morphological overlap between infections, lymphoma and non-infectious reactive lymphoproliferations in biopsies of patients with ID, clonality assessment can be helpful in predicting outcome (47) and guiding therapy (48). ID can cause complex proliferations of both T- and B-cells as a result of varying stimuli and transforming events, in which EBV plays an important role (49). This can result in oligoclonal expansions and subclones, which can develop into clonally related lymphomas (50). Since treatment is tailored to the immune status, the underlying trigger, the aggressiveness of the lymphoproliferation and whether it is a recurrent disease, it is important to unravel clonal relationships between simultaneous or subsequent biopsies, which can be facilitated by NGS-based clonality assessment.

### 3.5 Assessment of clonal relationship

In patients with synchronous or metachronous lymphomas, the treatment can depend on whether these lymphomas are clonally related. Comparison of the morphology and immunophenotype of the primary lymphoma and the relapse cannot always be used as a surrogate for clonal comparison, as clonally related relapses can show a different morphology or immunophenotype (51, 52) and clonally unrelated relapses can look similar to the primary lymphoma. In cHL, unrelated relapse can be suspected by a change of histologic subtype or an altered EBV association, but this is not absolute (53). Therefore, clonal comparison is an advised in lymphoma relapse with a long interval.

With conventional clonality analysis, the sizes of the clonal products in at least two targets are compared to assess clonal relationship. If a single clonal product is available for assessment the result remains uncertain (54), since two different rearrangements can coincidentally have the same size. Contrary, differences in peak sizes can be a result of somatic hypermutation within a clone, rather than a different clonal origin (55). NGS-based clonality analysis markedly improves the assessment of clonal relationship as it allows comparison of the actual sequence, enabling easy assessment of a clonal relationship even from a single target.

The incidence of second primary lymphomas is not well known since the studies are often small with a focus on specific types of lymphoma and with a selection for recurrences after a long interval. In some larger studies investigating diffuse large B-cell lymphoma (DLBCL) with a long interval between diagnosis and relapse up to 25% of relapsed DLBCL were actually unrelated (56–58).

In cHL it is even more difficult to investigate clonal relationship between primary and relapsed lymphoma due to the scarcity of tumor cells. A study of 20 patients with relapsed cHL showed 40% clonally unrelated tumors (53). In this study, the samples were enriched for tumor cells with laser microdissection, a laborious technique which is not suitable for routine diagnostics. NGS-based clonality analysis is more suitable to detect clonal products in cHL in comparison with conventional clonality analysis without laser microdissection (16, 59). This opens up possibilities to evaluate the clonal relationship in relapsed cHL in routine diagnostics.

### 3.6 Transformation and transdifferentiation

In patients with low-grade BCL who develop a high-grade lymphoma, it can be important for treatment decisions and assessment of prognosis to determine if the transformed lymphoma is indeed related to the low-grade lymphoma, or whether it is a *de novo* high-grade lymphoma. This has been most extensively studied in chronic lymphocytic leukemia (CLL) where a limited percentage of patients develops a secondary aggressive lymphoma, usually DLBCL. Transformation to cHL is infrequent. DLBCL development is more often clonally related, showing identical IG rearrangement to the CLL, than cHL (∼70% vs 40%) (60, 61). Patients with unrelated lymphoma have a better prognosis and are therefore treated differently than patients with clonally related lymphomas (62). The investigation of the clonal relationship is thus of clinical importance. As discussed above, the currently used clonality analysis has limitations in its ability to demonstrate clonal relationship, which can be overcome by NGS-based clonality.

In follicular lymphoma, transformation usually results in DLBCL or high-grade B-cell lymphoma with *MYC* and *BCL2* rearrangements (63). Transformation to cHL is rare (64). A diagnosis of transformed FL is usually made based on an assumed clonal relationship. Indeed, a clonal relationship between the initial FL and the transformation has only been established in small case series, but large studies are lacking (65–68).

In transdifferentiation, or lineage reprogramming, lymphoma cells acquire additional genetic aberrations leading to loss of B- or T-cell specific transcription factors. The resulting tumors show expression of a myeloid differentiation program. This causes a change of the morphology and phenotype from a lymphoma cell to a myeloid cell, most commonly resulting in a histiocytic or dendritic sarcoma (69). Since these sarcomas usually retain the original IG or TR rearrangements they can be clonally linked to the previous lymphoma.

### 3.7 TR repertoire analysis in patients with solid tumors and lymphoma

Immune escape represents an important mechanism in cancer development. Immune-checkpoint inhibition therapies directed against inhibitory checkpoint molecules such as PD-1 and CTLA-4 have become standard-of-care for several types of tumors, such as stage III or IV melanoma (70, 71), resectable lung cancer (72, 73), and colorectal cancer (74, 75). They exploit re-activation of T-cells that target neo-antigens arising from mutations in tumor cells. Analyzing the TR repertoire in a sample of interest can be performed using NGS (76). The dynamics of tumor and treatment related T-cell clones are an areas of intense research. In melanoma, breast and colon cancer, expanded T-cell clones are associated with response to immunotherapy treatment (77–79). In BCL, a restricted TR repertoire is found to be associated with poor outcome in DLBCL treated without immune-checkpoint inhibition (80) and in high grade B-cell lymphomas (81). Obviously, more studies are needed for different lymphoma types and treatments to understand the full potential of TR repertoire analysis in BCL.

## 4 Concluding remarks

The advent of NGS-based clonality analysis opens new possibilities for pathologists to define malignant lymphoproliferations in challenging clinical and histological situations and to discover clonal relationships between populations of lymphocytes in diverse infiltrates. B- and T-cell repertoire analysis in tissues in the context of immunodeficiency, autoimmune disease, lymphoma or solid tumors is a yet to be explored field with the potential to enable early detection of lymphoma development or prediction of therapy response.

## Data availability statement

The original contributions presented in the study are included in the article/supplementary material. Further inquiries can be directed to the corresponding author.

## Author contributions

PG and KH designed and coordinated the manuscript. PG, MvdB, AA, and KH wrote the manuscript, LK made the figures and edited the manuscript. All co-authors take responsibility for the integrity of the literature study and all co-authors critically revised the manuscript for important intellectual content. All authors contributed to the article and approved the submitted version.
